# Dual Atrioventricular Nodal Pathways Physiology: A Review of Relevant Anatomy, Electrophysiology, and Electrocardiographic Manifestations

**DOI:** 10.1016/s0972-6292(16)30711-2

**Published:** 2014-01-01

**Authors:** Bhalaghuru Chokkalingam Mani, Behzad B Pavri

**Affiliations:** Jefferson Medical College of Thomas Jefferson University, 925 Chestnut Street, Mezzanine, Philadelphia, PA19107

**Keywords:** dual AV nodal, arrhythmias, physiology, manifestations

## Abstract

More than half a century has passed since the concept of dual atrioventricular (AV) nodal pathways physiology was conceived. Dual AV nodal pathways have been shown to be responsible for many clinical arrhythmia syndromes, most notably AV nodal reentrant tachycardia. Although there has been a considerable amount of research on this topic, the subject of dual AV nodal pathways physiology remains heavily debated and discussed. Despite advances in understanding arrhythmia mechanisms and the widespread use of invasive electrophysiologic studies, there is still disagreement on the anatomy and physiology of the AV node that is the basis of discontinuous antegrade AV conduction. The purpose of this paper is to review the concept of dual AV nodal pathways physiology and its varied electrocardiographic manifestations.

The term "dual AV nodal pathways" is loosely used by many clinicians when analyzing electrocardiograms and telemetry strips. This term, to the novice physician, might suggest two, distinct anatomic structures related to the atrioventricular (AV) node, but often the true understanding of this entity is either missing or incomplete. The purpose of this manuscript is to review the anatomy, physiology, and electrophysiologic functional characteristics of the human AV node, and demonstrate the variety of electrocardiographic manifestations of what should correctly be described as "dual AV nodal pathways physiology". This review may be most helpful to physicians-in-training - interns, residents, and cardiology or pulmonary fellows; practicing internists with special interest in electrocardiography may also find this of value.

## Anatomy of the AV node

The AV node is part of the AV conduction axis. By virtue of its unique property of *decremental conduction*, the AV node delays the impulse arriving from the atria, thereby allowing the ventricles to stay in diastole, providing sufficient ventricular filling time. This property of decremental conduction also allows for protection of the ventricles from very rapid rates during atrial fibrillation. Though there were numerous researchers who identified the presence of an electrical connection between the atria and the ventricles, the physical existence of the AV node was definitively proven by Tawara. [1,2] 

**Figure 1:**
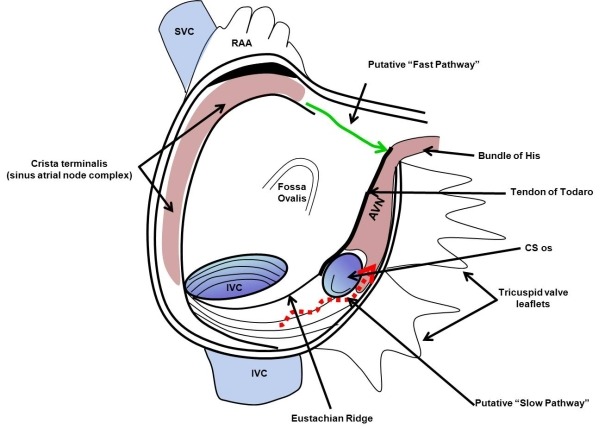
Schematic representation of the interior of the right atrium, as viewed in the right anterior oblique projection. The transmission of impulse from the sinoatrial node over the "fast pathway" (green arrow) and over the "slow pathway" (red dashed arrow) to the AV node is depicted.

The compact AV node is located at the base of the atrial septum in the triangle of Koch [3] (Figure 1). The triangle is visualized on the right side of the endocardial surface of the interatrial septum, and is formed anteriorly by the insertion of the septal leaflet of the tricuspid valve and posteriorly by the fibrous tendon of Todaro [4]. The apex of the triangle is formed by the junction of these two boundaries and the base is formed by the superior lip of the orifice of the coronary sinus [5]. The AV node can be considered to be made up of two zones, transitional and compact. The transitional zone consists of "transitional cells", so called because they are intermediate in morphology and function between the compact nodal cells and the atrial myocytes. This zone of transitional cells envelops the compact AV node, serving as the connection between the surrounding atrial myocardium and the compact node [6].

The compact zone measures 5-7 mm in length and 3-4 mm in width and is located in the triangle of Koch. The compact AV node gives rise to three posterior extensions: one in the direction of the coronary sinus along the tricuspid annulus (the putative "slow pathway"), a second in the anterior portion of the triangle of Koch near the compact portion of the AV node (the putative "fast pathway"), and the third in the direction of the mitral annulus (the left atrial extension) [7]. The anatomic separation of about 15 mm [8] between the anterior (fast) and posterior (slow) approaches is what allows safe ablation of the putative "slow pathway" for the treatment of AV nodal reentrant tachycardia without the creation of complete heart block.

## Electrophysiology of the AV node

The mechanism underlying the unique property of decremental conduction is not fully understood, although two hypotheses have been put forth. The first is referred to as the decremental driving force hypothesis and suggests that conduction along the AV node may change in such a way that the propagating "action potential becomes progressively less effective as a stimulus to the unexcited portion of the fiber ahead of it" [9]. The second hypothesis is referred to as the electrotonic transmission hypothesis,[10] and states that the driving voltage is constant, but that inexcitable microscopic segments cause "stagnation" between different zones of the AV node. Recent studies assessing expression of gap junction proteins, specifically connexin 43 (Cx43), shows concordance of Cx43 distribution along anatomically defined AV nodal structures [11].

The wave front of atrial activation engages the AV node at multiple locations. The dual AV nodal system involves two separate approaches to the compact AV node, the so-called fast and slow "pathways". As is true for most excitable tissue, the (anteriorly located) "fast pathway" demonstrates the greater conduction velocity, but takes longer to recover from excitation; i.e., *the fast pathway has the longer refractory period*. The (posteriorly located) "slow pathway" has the slower conduction velocity, but recovers faster from prior depolarization, i.e., *the slow pathway has the shorter refractory period*. Elegant studies have shown that the posterior extension of the AV node provides the anatomic basis for all the electrophysiologic properties ascribed to the "slow pathway" [12]. Although not discretely identifiable as separate anatomic structures, these inputs result in a highly heterogeneous engagement of the AV node as shown by optical mapping studies [13]. These approaches form the basis for "dual AV nodal pathways" physiology.

**Figure 2:**
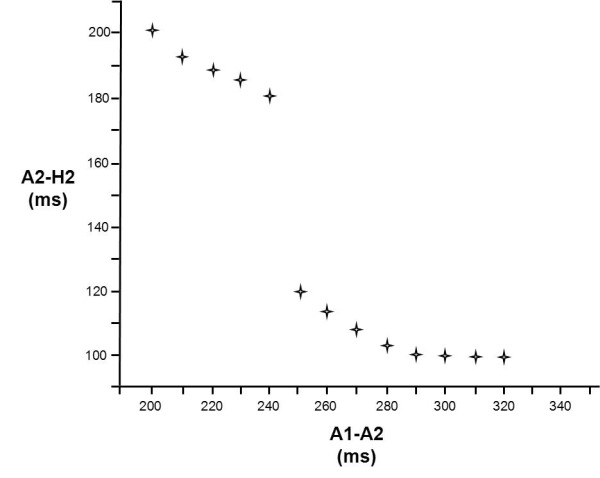
This graph shows a plot of prematurity versus AV nodal conduction time. A1: last paced impulse of the drive train; A2: premature atrial impulse. The X axis represents the time interval between the last beat of the drive train and the premature atrial impulse. The Y axis represents the conduction time between the premature atrial impulse and the resulting His bundle deflection.

The electrophysiologic definition of dual pathways physiology in the electrophysiology laboratory depends on the demonstration of *discontinuous antegrade AV nodal conduction* in response to atrial programmed electrical premature stimulation. This involves delivery of a train of eight impulses (A1) at a fixed cycle length, followed by a single premature impulse (A2). The conduction time of this premature impulse (A2) through the AV node (the AH interval) is measured, as the degree of prematurity is gradually increased in 10 ms decrements. Decremental conduction of A2 through the AV node results in progressive and gradual prolongation of nodal conduction time with increasing prematurity. An abrupt increase in AV nodal conduction time of ≥50 ms in response to a decrement of 10 ms is the (somewhat arbitrary) definition of dual AV nodal pathways physiology, often referred to as a "jump". Multiple abrupt increases ("jumps") are not uncommon, and may reflect multiple functional "pathways". A typical plot of prematurity versus AV nodal conduction time with a single "jump" is shown in Figure 2.

There are occasional patients where AV nodal conduction times continuously prolong (without a discrete "jump") in the face of increasing atrial prematurity, until retrograde conduction starts over the "fast pathway" and atrioventricular nodal reentrant tachycardia is induced. The exact mechanism of this observation is unclear, but may relate to minimal differences in the antegrade refractory periods of the fast and slow "pathways". Finally, the mere demonstration of dual AV nodal pathways physiology does not automatically imply that AV nodal reentrant supraventricular tachycardia will always inducible; in fact, many patients who undergo electrophysiology study will show anterograde "jumps" with programmed atrial stimulation, even single "echo" beats, but will not have sustained tachycardia. There needs to be sufficiently slow conduction over the anterograde "slow pathway, and a sufficiently quick recovery (short refractory period) of the retrograde fast pathway, for AV nodal reentrant tachycardia to sustain.

Although functional properties may be related to anatomic/electrophysiologic differences, the profound influence of autonomic nervous control on the AV node cannot be overstated. AV nodal conduction time and refractoriness are exquisitely controlled by sympathovagal balance. Sympathetic stimulation shortens conduction time and refractoriness, whereas vagal stimulation provides the opposite effect. These observations have been used to control ventricular rate in atrial fibrillation by stimulation of post-ganglionic vagal fibers near the ostium of the coronary sinus in animal [14] and human [15,16], models. At rest, increasing atrial rates (as during atrial pacing) result in decremental AV nodal conduction, as discussed above. However, during exercise, increasing sinus rates are accompanied by *shortening* of AV nodal conduction times, as manifested by PR interval shortening.

Antiarrhythmic drugs may preferentially affect slow or fast pathway conduction, but these effects are not consistent, and are influenced by the presence of underlying conduction system disease. Blockade of the sympathetic and vagal influences by administration of propranolol and atropine, respectively, renders the majority of AVNRT non-inducible [17]; this observation supports the "functional" quality of dual AV nodal pathways physiology.

## Modulation of dual AV nodal pathways physiology

### Effect of Adenosine on dual pathways physiology

Low doses of adenosine administered in sinus rhythm or during atrial pacing preferentially slow conduction in the fast pathway (mean dose required for fast pathway block was 2.7±3.0 mg), resulting in manifest engagement of the slow AV nodal approach; block in the slow pathway required a higher dose (mean 7.2±4.7 mg) [18]. Low-dose adenosine can serve as a non-invasive diagnostic test of dual AV nodal physiology. Higher doses block conduction in both slow and fast approaches, and adenosine is clinically used to terminate reentrant arrhythmias that involve the AV node. Bedside administration of adenosine can help make the diagnosis of dual AV nodal pathways physiology.[19,20]

### Effect of Vagus nerve stimulation on dual pathways physiology

**Figure 3:**
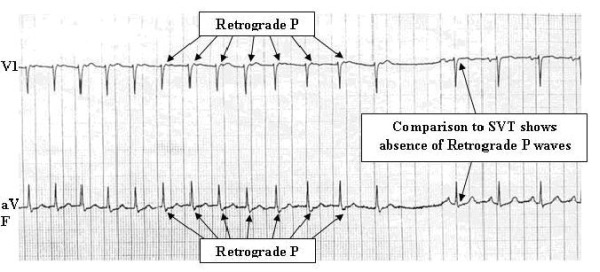
Typical AVNRT terminated by carotid sinus massage. The RP interval is short (less than 100ms) favoring typical slow-fast AVNRT as the SVT mechanism. The retrograde P wave is inscribed immediately following the QRS complex (creates the appearance of a broader "S" wave in lead aVF), and may be missed during a cursory review; however, comparison to the QRS complex in sinus rhythm after SVT termination (right side of tracing) allows immediate recognition of the P wave location during SVT. Note that the SVT ends with a retrograde P wave, and is consistent with block in the anterograde slow pathway as the site of SVT termination.

Fisch et al proposed that the changes in autonomic influence could be the potential trigger in shifting conduction from the fast to the slow pathway, resulting in sudden changes in PR interval. Chiou et al showed that that increase in vagal tone preferentially prolonged the effective refractory period of the fast pathway as compared to slow pathway [22. However, carotid massage will often stop typical AVNRT in the anterograde slow pathway (see Figure 3).

### Effect of parasympathetic blockade vs. adrenergic stimulation on dual pathways physiology

Stellbrink et al tested the comparative efficacy of parasympathetic blockade vs. adrenergic stimulation on inducibility of AVNRT at the time of EP testing [23]. Atropine (0.01 mg/kg) was compared to isoproterenol (0.5-1.0 mcg/kg/min infusion) prior to ablation. Atropine reduced inducibility, whereas isoproterenol increased inducibility of AVNRT, mainly by accelerating slow pathway conduction velocity.

### Effect of beta-adrenergic receptor blockade on dual pathways physiology

Esmolol was shown to have quantitatively greater effect on antegrade refractoriness of the fast pathway as compared to the slow pathway [24].

## ECG manifestations of dual AV nodal pathways physiology

Dual pathway physiology can be electrocardiographically "silent" with no manifestations whatsoever. The prevalence of dual pathways physiology is variably reported as being demonstrable in 10 to 35% of normal people [25-28] including children [29], but may become less common with aging [30]. A multitude of electrocardiographic manifestations [19,31,32] may be explained by dual pathways physiology (see Table 1).

### Rapid ventricular rates during atrial fibrillation

**Figure 4:**
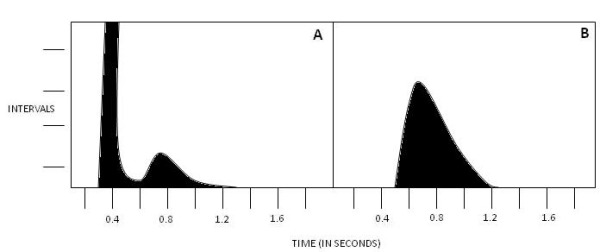
Histogram A Shows Bimodal RR interval distribution in a patient with atrial fibrillation before AV node modification. Histogram B shows unimodal RR interval distribution after AV node modification due to slow pathway ablation which has eliminated the peak with the shorter RR intervals (modified with permission from Tebbenjohanns J, Schumacher B, Korte T, Niehaus M, Pfeiffer D. Bimodal RR interval distribution in chronic atrial fibrillation: impact of dual atrioventricular nodal physiology on long-term rate control after catheter ablation of the posterior atrionodal input. J Cardiovasc Electrophysiol. 2000;11(5):497-503).

Based on its shorter refractory period (see above), it is believed that the shortest R-R intervals (the fastest rates) during atrial fibrillation represent conduction over the "slow pathway" of the AV node; ablation of the slow pathway region in patients with atrial fibrillation results in significant slowing of the ventricular rates [33]. This is reflected in a bimodal distribution of R-R intervals on pre-ablation Holter recordings, which is replaced by a unimodal pattern after ablation of the slow pathway [34], as shown in Figure 4.

In fact, the presence of a bimodal R-R interval plot prior to ablation predicts a better outcome after ablation of the slow pathway region, as compared to patients with a unimodal R-R interval distribution [35].

### Atrioventricular Nodal Reentrant Tachycardia (AVNRT)

AVNRT is the commonest form of regular supraventricular tachycardia in humans, and presents as regular narrow complex tachycardia on the ECG. AVNRT can be typical (also referred to as the common form) or atypical (the uncommon form) depending on the location of the atrial deflection between consecutive QRS complexes. This is thought to reflect direction of reentrant excitation with the AV nodal circuit [36].

**Figure 5:**
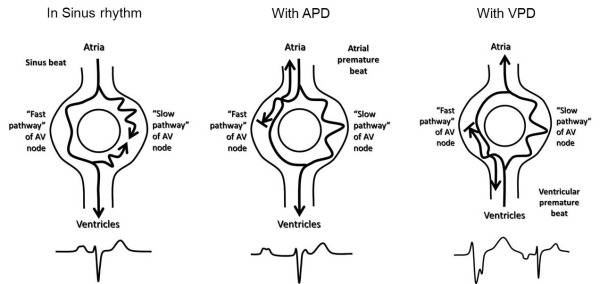
Model of dual AV nodal pathways physiology in sinus rhythm, with an atrial premature beat (APD) which initiates typical "slow-fast" AVNRT, and with a ventricular premature beat (VPD) which initiates atypical "fast-slow" AVNRT. See text for details.

Typical AVNRT or the "slow-fast" type is the commonest (>80%) form of AVNRT. The earliest site of retrograde atrial activation in this type is seen in the region of the fast pathway near the apex of the triangle of Koch. It is usually initiated by an atrial premature depolarization which, by virtue of prematurity, finds the fast pathway refractory and conducts to the ventricles via the slow pathway (see Figure 5), middle panel); the surface ECG records a prolonged PR interval. If sufficient time has lapsed to allow recovery of the fast pathway, the impulse can conduct rapidly up the fast pathway, resulting in a typical AV nodal "echo" beat; the surface ECG records a short RP interval. If this pattern perpetuates, AVNRT is initiated. Typical AVNRT can be initiated by single or multiple atrial premature complexes.

**Figure 6:**
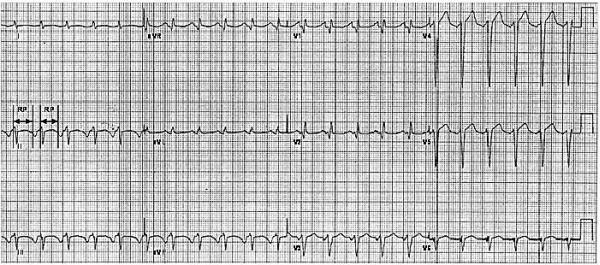
Atypical AVNRT; the RP interval is long (about 310 milliseconds) favoring atypical fast-slow AVNRT as the SVT mechanism. The retrograde P wave is inscribed immediately before the QRS complex and is superimposed on the T wave.

Atypical or the "fast-slow" type of AVNRT is relatively uncommon (about 5%). The earliest site of retrograde atrial activation in this type is seen in the region of the slow pathway near the ostium of the coronary sinus. Atypical AVNRT (see Figure 6) is often initiated by a premature ventricular complex that conducts retrogradely through the slow pathway to the atrium, having found the fast pathway inexcitable (see Figure 5, right panel); the surface ECG records a long RP interval (see Figure 5). If retrograde conduction is sufficiently slow, the fast pathway may recover sufficiently to carry the excitation back to the ventricles, resulting in an atypical AV nodal "echo" beat; the surface ECG records a short PR interval. The earliest site of atrial activation is near the ostium of the coronary sinus. Less often, this form of AVNRT can also be initiated by an atrial premature complex which conducts to the ventricles over the fast pathway, and conducts back to the atrium over the slow pathway.

Other atypical variants of AVNRT (~14%) [32] are also described, including slow-slow, fast-intermediate and slow-intermediate forms which use functionally and or anatomically distinct fast, slow or intermediate pathways to induce tachycardia. However, the site of earliest retrograde atrial activation in these forms is more variable [37]. Further studies are required to understand why certain tissues within the AV node complex demonstrate different conduction velocities, but one potential explanation is the heterogeneous distribution of Connexins within the AV node [38].

### Post-PVC PR interval prolongation - concealed conduction

**Figure 7:**
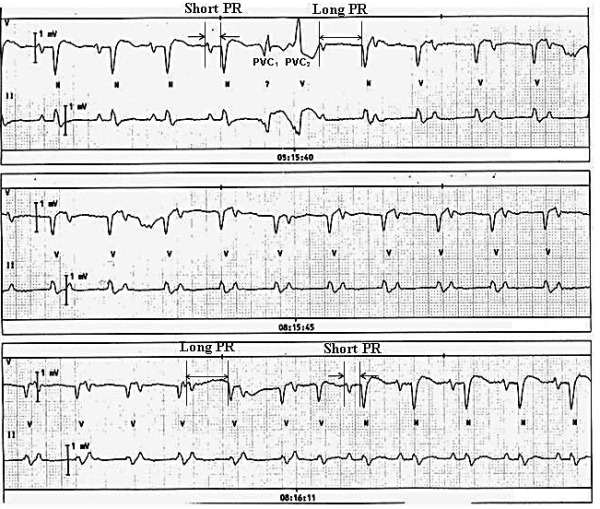
Post-PVC PR prolongation. The 5th and 6th beats in the top tracing are premature ventricular contractions (PVC1 and PVC2); PVC2 conducts retrogradely into the fast pathway of the AV node, and renders it refractory. The next P wave conducts anterogradely via the slow pathway of the AV node resulting in significant PR prolongation. Anterograde slow pathway conduction persists due to concealed retrograde conduction into the fast pathway (so called concealed "linking"), continues until the middle of the bottom tracing, where anterograde conduction through the normal fast pathway resumes.

Concealed conduction is a phenomenon that describes partial penetration of an impulse into a given tissue (e.g., the AV node), but can only be inferred by the behavior of the subsequent impulse that conducts through the same tissue [39]. (See Figure 7) Concealed conduction can block the dual pathways physiology from being set into motion by prolonging the refractory period of the slow pathway.[29]

### Two families of PR Interval

**Figure 8:**
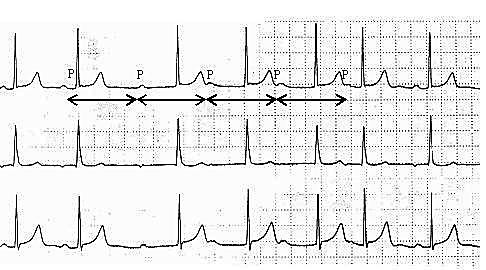
A spontaneous change in PR interval during regular sinus rhythm at 65 beats per minute is noted. There are two "families" of PR intervals: one group of PR intervals measure about 220 ms (representing conduction over the "fast pathway"), and the other group is about 520 ms (representing conduction over the "slow pathway"). Note that the PR interval over the "fast pathway" is abnormally prolonged.

ON occasion, dual AV nodal physiology can present as normal sinus rhythm with subtle ECG changes which manifest as a abrupt lengthening or shortening of the PR interval (see Figure 8). The shorter PR interval represents conduction over the fast pathway (normal) and the longer PR represents conduction over the slow pathway. The shift in conduction from fast to slow pathway can occur spontaneously or can be provoked by an atrial premature complex or a ventricular premature complex. Similarly the conduction through the slow pathway can be terminated by an atrial premature complex, a ventricular premature complex or even a short run of atrial tachycardia. Uncommonly sinus rhythm with PR alternans can occur in which a long and short PR interval alternate with each other.[17] It can also manifest as two families of PR intervals that signify presence of underlying dual AV nodal physiology.[19,30]

Interestingly, patients with two families of PR interval almost never show reentrant AVNRT; this is related to extensive "fast pathway" disease, which does not allow retrograde conduction.

### Two families of RP Interval

**Figure 9:**
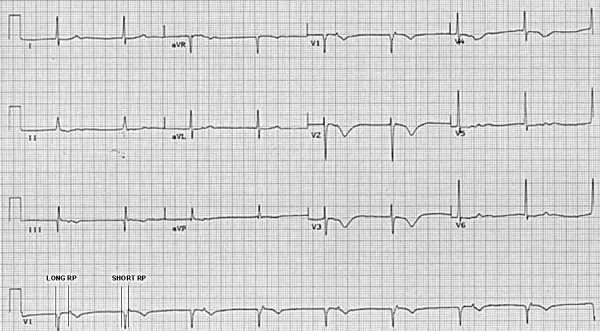
This ECG shows a junctional (narrow complex) rhythm at 52 bpm with 1:1 junction-to-atrium conduction with retrograde P waves. However, the RP interval alternates between 80 ms and 180 ms, consistent with "dual AV nodal pathways" physiology. The QTc interval is abnormally prolonged.

Rarely, the same physiology described above in the anterograde direction may be seen in the retrograde direction as well. This would manifest as junctional or ventricular rhythms with 1:1 retrograde conduction, but with 2 families of RP interval (see Figure 9).

### Pseudo-Interpolation

**Figure 10:**
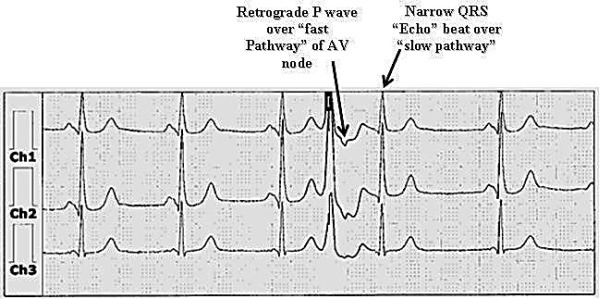
Pseudo-interpolation. A premature ventricular complex conducts to the atrium through the "fast pathway" producing a retrograde P wave. This P wave then conducts through the slow pathway with a prolonged PR interval, producing a ventricular "echo" beat, mimicking true interpolation.

In fact, one of the earliest manifestations of dual pathways physiology was shown by Kistin in 1962, when he noted that apparent interpolation of PVCs was in fact due to AV nodal "echo" beats [40]. Therefore, single AV nodal echo beats can mimic interpolation (see Figure 10).

### Double ventricular response to a single atrial depolarization - "Double Fire"

**Figure 11:**
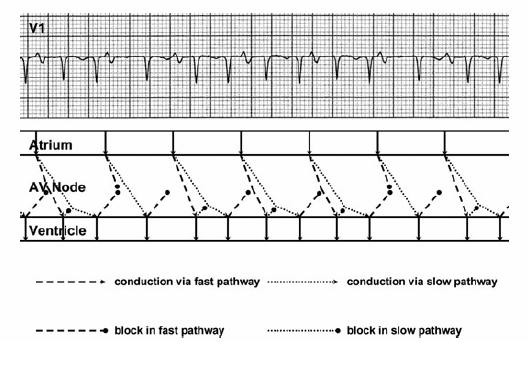
The rhythm strip is lead V1 of a surface electrocardiogram. A ladder diagram demonstrated the proposed mechanism for 1:2 AV conduction via dual AV nodal pathways. Concealed retrograde conduction in both fast and slow pathways led to a pseudo-Wenckebach pattern. The Wenckebach cycle length of the fast pathway during the electrophysiology study was 320 ms, significantly shorter than the sinus cycle length here. Reprinted with permission from Wang NC, Razak EA, Jain SK, Saba S. Isoproterenol facilitation of slow pathway ablation in incessant dual atrioventricular nodal nonreentrant tachycardia. Pacing Clin Electrophysiol 2012;35(2):e31-4.

One of the rarer ECG manifestations of dual pathways physiology, a double ventricular response to a single atrial impulse is due to the anterograde conduction of the impulse in both the fast and the slow pathways (see Figure 11). This requires that slow pathway conduction must be slow enough to allow His-Purkinje tissue to recover excitability after being depolarized by the first excitation over the fast pathway. In addition, unidirectional retrograde block in the both pathways is essential for this to occur [41]. Double ventricular responses arising from one atrial impulse may result in tachycardia that does not utilize a reentrant circuit, and has been referred to as "double fire" or non-reentrant tachycardia, first described by Csapo G as early as 1979 [42]. Since then, double fire tachycardia and tachycardia-related cardiomyopathy that reverses with ablation of the slow pathway have been well described [43].

## Summary

This review discusses the fundamental anatomy and physiology underlying dual AV nodal pathway function, and the resulting ECG manifestations. The astute physician will learn to recognize the myriad ECG manifestations of dual pathways physiology, and offer appropriate investigations and therapeutic decisions for optimal patient care. Recognizing dual AV nodal pathways requires a thorough understanding of AV nodal physiology.

## Figures and Tables

**Table 1:**
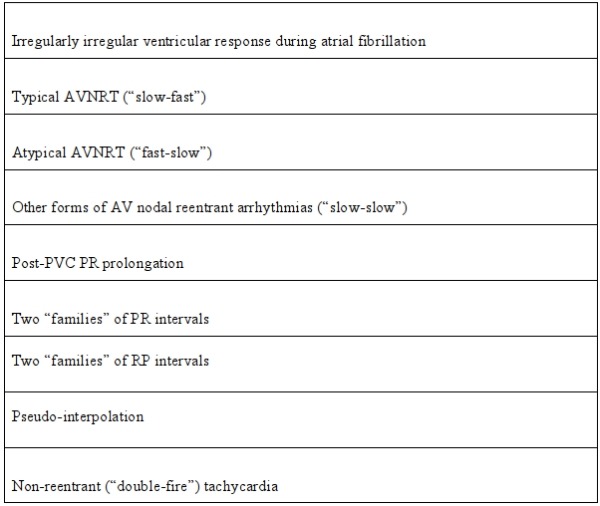
ECG Manifestations of Dual AV Nodal Pathways Physiology
